# Shorter laboratory turnaround time is associated with shorter emergency department length of stay: a retrospective cohort study

**DOI:** 10.1186/s12873-022-00763-w

**Published:** 2022-12-21

**Authors:** Bram E. L. Vrijsen, Saskia Haitjema, Jan Westerink, Cornelia A. R. Hulsbergen-Veelken, Wouter W. van Solinge, Maarten J. ten Berg

**Affiliations:** 1 Department of Internal Medicine, Division Internal Medicine and Dermatology, University Medical Center Utrecht, Utrecht University, Utrecht, the Netherlands; 2 Central Diagnostic Laboratory, Division Laboratories, Pharmacy and Biomedical Genetics, University Medical Center Utrecht, Utrecht University, Utrecht, the Netherlands

**Keywords:** Clinical laboratory techniques, Hospital emergency services., Length of stay.

## Abstract

**Background:**

A longer emergency department length of stay (EDLOS) is associated with poor outcomes. Shortening EDLOS is difficult, due to its multifactorial nature. A potential way to improve EDLOS is through shorter turnaround times for diagnostic testing. This study aimed to investigate whether a shorter laboratory turnaround time (TAT) and time to testing (TTT) were associated with a shorter EDLOS.

**Methods:**

A retrospective cohort study was performed, including all visits to the emergency department (ED) of an academic teaching hospital from 2017 to 2020 during which a standardized panel of laboratory tests had been ordered. TTT was calculated as the time from arrival in the ED to the ordering of laboratory testing. TAT was calculated as the time from test ordering to the reporting of the results, and was divided into a clinical and a laboratory stage. The outcome was EDLOS in minutes. The effect of TTT and TAT on EDLOS was estimated through a linear regression model.

**Results:**

In total, 23,718 ED visits were included in the analysis. Median EDLOS was 199.0 minutes (interquartile range [IQR] 146.0–268.0). Median TTT was 7.0 minutes (IQR 2.0–12.0) and median TAT was 51.1 minutes (IQR 41.1–65.0). Both TTT and TAT were positively associated with EDLOS. The laboratory stage comprised a median of 69% (IQR 59–78%) of total TAT.

**Conclusion:**

Longer TTT and TAT are independently associated with longer EDLOS. As the laboratory stage predominantly determines TAT, it provides a promising target for interventions to reduce EDLOS and ED crowding.

## Background

Emergency department length of stay (EDLOS) is an important benchmark of the quality of care in the emergency department (ED) [1]. EDLOS is affected by many factors, both patient-related and organizational. Generally, more complex and acute patients, who generally require more extensive diagnostics, have longer EDLOS [2, 3]. Organizational factors that increase EDLOS include a shortage of beds leading to hospital transfer, evaluation by medical students or trainees, and sequential specialist consultations [4, 5]. Extended lengths of stay lead to crowding in the ED, which in turn is associated with worse outcomes, including death [6–8]. In a study on patients with severe pneumonias, performed during the covid-19 pandemic, EDLOS was an independent risk factor for in-hospital mortality (odds ratio 1.84 for EDLOS in hours) [9]. Given the increasing age and complexity of patients presenting to the ED, long EDLOS is a growing concern [10]. Therefore it is important to look for interventions that shorten EDLOS.

A potential determinant of the EDLOS that has been relatively understudied is the laboratory testing turnaround time (TAT) [11, 12]. A shorter TAT results in the clinician, and thus the patient, having earlier access to the test results, which play an important role in many medical decisions [13].

One solution to shorten TAT is point-of-care testing, in which laboratory tests are performed at the patient’s bedside [14]. In the ED, point-of-care testing has been shown to lead to shorter length of stay in some studies, [15–18] but not in all [19–21]. Studies also show conflicting results on the effect of point-of-care testing on hospital admission rates in the same patient groups [18, 19]. These conflicting results may be caused by point-of-care testing only being available for a limited number of tests, such as cardiac markers, blood gases and certain electrolytes. Often, additional laboratory tests at the central laboratory are required, which might prolong the EDLOS. In one trial, 94.7% of patients who had point-of-care testing done still required additional testing from the central laboratory [22].

Therefore, the TAT of regular laboratory tests at the central laboratory may prove a more promising target for shortening the EDLOS. Many studies on TAT have focused on the laboratory’s perspective, but from the perspectives of the patient and the clinician, what matters is not only the time the laboratory needs to generate the results, but also the time required to send the samples to the laboratory and even the time it takes the clinician to decide to order laboratory testing in the first place, which we dubbed the time to testing (TTT). To our knowledge the latter has not been studied in relation to EDLOS.

Consequently, this study was set up to investigate whether a shorter TAT and TTT were associated with a shorter EDLOS.

## Methods

### Patient selection

This is a single center retrospective cohort study performed in the University Medical Center Utrecht (UMC Utrecht), an academic teaching hospital in the Netherlands, with around 20,000 ED visits per year. The study period ran from January 2017 until January 2020. The study population included all ED visits of adult patients for whom a standard panel of laboratory tests was ordered (pre-defined in our electronic health record as the “internal medicine lab”) through the order management module of the electronic health record. This standard panel comprises the following 14 tests: hemoglobin, thrombocyte count, leucocyte count, sodium, potassium, urea, creatinine, alkaline phosphatase, gamma glutamyltransferase, alanine transaminase, aspartate transaminase, lactate dehydrogenase, glucose, and C-reactive protein. Other tests can be added to this panel, for instance cardiac markers in patients with suspected myocardial infarction. Only visits in which these standard tests were ordered were included to prevent confounding by indication.

If the standard panel was ordered more than once during an ED visit, the first order was used for the analysis. Visits in which not all of the ordered tests of the standard panel were actually performed were excluded from the analysis. If patients visited the ED more than once in the study period all eligible visits were included in the study and were seen as individual events.

In the ED, the nurses are responsible for the venipuncture and the transportation of the samples to the laboratory by pneumatic tube.

### Measures and outcomes

The primary outcome was the EDLOS, defined as the time from arrival in the ED to either discharge, admittance, transfer elsewhere, or death, in minutes.

The TTT was calculated as the time in minutes between the patient’s arriving in the ED and the ordering of laboratory tests. The TAT was calculated as the time in minutes between the time the laboratory tests were ordered at the ED and the time the last result of all tests in the standard panel was reported in the electronic health record.

**Fig. 1:**
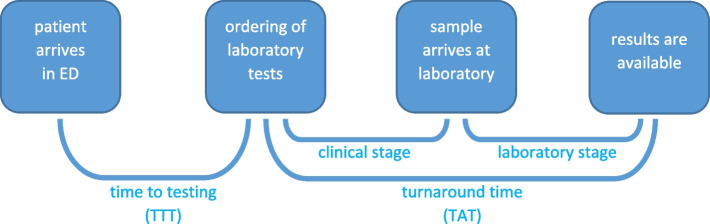
Time to testing (TTT) and turnaround time (TAT). ED: emergency department. All times are reported in minutes

Furthermore, the TAT was divided into a clinical stage (the time from the ordering of the laboratory tests to the arrival at the laboratory) and a laboratory stage (the time from the arrival of the sample at the laboratory to the results becoming available in the electronic health record). The different times are represented schematically in Fig. 1.

Additionally, data were collected on the date of the visit, the patient’s age and sex, the time of arrival at the ED (recorded as being between 8:00 and 16:59 (day time shift), between 17:00 and 00:59 (evening shift) or between 01:00 and 07:59 (night shift), the Emergency Severity Index level [23], which is used as the triage system in the ED, the number of out-of-range laboratory test results in the panel, the daily total number of ED visits, the destination after the ED visit (discharge home, admittance to hospital, transfer to another facility, or deceased) and the specialty treating the patient at the ED (grouped into medicine, surgery, and other specialties including psychiatry, rehabilitation medicine, pain management, and radiotherapy).

The time of day and the date were used to account for potential confounding due to variations in the laboratory workload and ED crowding both over time and at different times of the day. The Emergency Severity Index level, the specialty, the destination after the ED visit and the number of out-of-range tests were included as proxies for the acuity level, which was identified as a confounder in previous studies [11, 12].

### Data acquisition

All determinants were collected by two of the authors through the Utrecht Patient Oriented Database (UPOD) and checked for quality by a third. UPOD is an infrastructure of relational databases that automatically retrieve data from the hospital information system on patient characteristics, hospital discharge diagnoses, medical procedures, medication orders and laboratory tests for all patients treated at the UMC Utrecht since 2004. UPOD data acquisition and management is in accordance with current regulations concerning privacy and ethics. The structure and content of UPOD have been described in more detail elsewhere [24]. All measures regarding ED arrival and discharge and the logistics of the laboratory tests are automatically time stamped in the hospital information system.

### Statistical analysis

Baseline characteristics were reported. Means and standard deviations (SD) were calculated for normally distributed data, and medians and interquartile ranges (IQR) for non-normal data. Visits with data suspected to be incorrect (e.g. incorrect time stamps due to the change from daylight savings time) were excluded. In order to handle erratic outliers caused by administrative errors, the data set was trimmed to exclude the top and bottom 0.5% values for the EDLOS and the clinical and laboratory stages of the TAT.

The effect of the total TAT and TTT on the EDLOS was estimated by using linear regression, both in a univariate model as in a multivariate model, controlling for the other variables mentioned above.

The regression model gives estimates for the increase in EDLOS in minutes per 1 minute increases in TAT and TTT, as well as for changes in the other variables. A *p*-value of < 0.05 was considered significant. A sensitivity analysis was performed by performing the linear regression on the full dataset including the trimmed values. Furthermore, we calculated how much of the total TAT consisted of the clinical and laboratory stages. As a post hoc analysis, we compared the TAT for the different times of day, which was tested using the Kruskal-Wallis test. All analyses were performed in R version 4.0.3.

## Results

There were 24,727 eligible visits to the ED during the study period, out of a total of 39,992 visits during which any laboratory tests were ordered. In 290 cases, not all laboratory tests that had been ordered were actually performed, and these were excluded. A further 6 patients were excluded because one of the time points in their ED visit fell in the transition period from summer time to winter time, which led to ambiguous turnaround times.

Trimming the data set to deal with outliers led to the exclusion of 713 cases (2.9%), which left 23,718 visits for the analysis.

The mean age of the included patients was 58.4 years (SD 17.8), and 47% of patients were female. The majority of visits (87%) were treated by medicine specialties. About half of the visits (53%) resulted in admission to the hospital. The median TTT was 7.0 minutes (IQR 2.0–12.0) and the median total TAT was 51.1 minutes (IQR 41.1–65.0). The median EDLOS was 199 minutes (IQR 146.0–268.0). All the baseline characteristics of the patients in the analysis are provided in Table 1.

**Table 1:** Baseline characteristics

total number of ED visits	23,718
age (in years; mean + SD)	58.4 ± 17.8
female sex	11,041 (47%)
specialty	
medicine	20,551 (87%)
surgery	3098 (13%)
other	69 (< 1%)
time of day	
8:00–16:59	14,279 (60%)
17:00–0:59	7510 (32%)
1:00–7:59	1929 (8%)
number of daily patients (mean + SD)	55.9 ± 8.7
number of abnormal tests (median + IQR)	5.0 (3.0–7.0)
destination	
home	10,713 (45%)
admission	12,524 (53%)
transfer to another facility	410 (2%)
death	71 (< 1%)
ESI triage level	
level 1	644 (3%)
level 2	6401 (27%)
level 5	23 (< 1%)
not scored	27 (< 1%)
TAT (in minutes; median + IQR)	51.1 (41.1–65.0)
TTT (in minutes; median + IQR)	7.0 (2.0–12.0)
EDLOS (in minutes; median + IQR)	199.0 (146.0–268.0)

All variables are absolute numbers and percentages (%) except where otherwise specified.

*ED* emergency department; *SD* standard deviation; *IQR* interquartile range; *ESI* Emergency Severity Index [23]; *TAT* turnaround time; *TTT* time to laboratory test ordering; *EDLOS* emergency department length of stay

After adjustment for the other co-variables in the model, a 1 minute increase in the TTT led to an increase in the EDLOS of 0.56 minutes (95% CI 0.50–0.62) while a 1 minute increase in TAT led to an increase in the EDLOS of 0.32 minutes (95% CI 0.28–0.37). Furthermore, age, female sex, the number of out-of-range laboratory tests, ED visits during office hours, the daily total number of patients in the ED, all but the highest Emergency Severity Index level, and being admitted to hospital or transferred to another facility were also associated with a longer EDLOS (Table 2). There were no relevant interactions between the variables in the model, so these were left out of the final model. The adjusted R^2^ of the model was 10%. Performing the linear regression on the untrimmed data set did not change the results.

**Table 2:** Determinants of EDLOS

	Change in EDLOS in minutes^a^ (univariate) (95% CI)	Change in EDLOS in minutes^a^ (multivariate) (95% CI)	*p*-value (multivariate)
TAT (in minutes)	0.50 (0.45–0.54)	0.32 (0.28–0.37)	< 0.001
TTT (in minutes)	0.50 (0.43–0.56)	0.56 (0.50–0.62)	< 0.001
time from the start of the study period (in days)	0.003 (− 0.001–0.007)	0.002 (− 0.002–0.006)	0.26
age (in years)	0.29 (0.22–0.36)	0.12 (0.05–0.19)	< 0.001
female sex	4.1 (1.7–6.5)	4.9 (2.6–7.2)	< 0.001
specialty			
medical	-^b^	-^b^	–
surgical	16.7 (13.1–20.2)	14.0 (10.6–17.4)	< 0.001
other	−36.4 (−58.7 – − 14.0)	−27.6 (− 49.1 – − 6.1)	0.01
time of day			
8:00–16:59	-^b^	-^b^	–
17:00–0:59	−24.9 (−27.5 – −22.3)	−20.7 (−23.3 – − 18.2)	< 0.001
1:00–7:59	− 34.6 (− 39.1 – − 30.2)	−25.7 (− 30.1 – − 21.4)	< 0.001
number of daily patients	0.28 (0.15–0.42)	0.20 (0.07–0.34)	0.003
number of abnormal tests	5.2 (4.87–5.6)	4.8 (4.4–5.3)	< 0.001
destination			
home	-^b^	-^b^	–
admission	11.2 (8.8–13.6)	6.2 (3.7–8.7)	< 0.001
transfer to another facility	97.2 (88.0–106.5)	90.0 (81.1–98.9)	< 0.001
death	13.0 (−8.8–34.9)	53.2 (31.5–74.9)	< 0.001
ESI triage level			
level 1	-^b^	-^b^	–
level 2	49.9 (42.3–57.5)	65.2 (57.7–72.7)	< 0.001
level 3	66.2 (58.8–73.6)	75.4 (68.0–82.8)	< 0.001
level 4	54.4 (45.6–63.1)	63.8 (55.1–72.5)	< 0.001
level 5	24.3 (−14.7–63.3)	22.1 (− 15.4–59.6)	0.25
not scored	8.6 (− 27.5–44.7)	12.0 (− 22.6–46.6)	0.50

^a^ The change in EDLOS is the change in minutes per one unit increase in the explanatory variable for continuous explanatory variables (minutes for TAT and TTT, days for the time from the start of the study period, years for age, and number for daily patients and abnormal tests. For categorical explanatory variables, it is the change in minutes compared to the reference category

^b^ Reference category. EDLOS: emergency department length of stay; CI: confidence interval; TAT: turnaround time; TTT: time to testing; ESI: Emergency Severity Index [23].

**Fig. 2:**
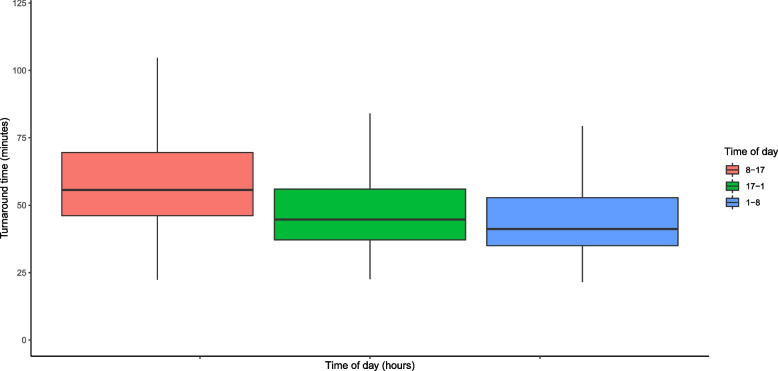
Laboratory turnaround times per time of day

The total TAT was mostly driven by the laboratory stage, which comprised a median of 69% (IQR 59–78%) of the total laboratory turnaround time. TAT was generally shorter in the evenings and nights (Fig. 2). The median TAT was 55.6 minutes (IQR 46.1–69.5) during office hours, 44.7 minutes (IQR 37.1–60.0) in the evenings and 41.2 minutes (IQR 35.0–52.8) in the nights (*p* <  0.001).

## Discussion

This study found that a 1 minute increase in TTT leads to a 0.56 minute increase in EDLOS and that a 1 minute increase in TAT leads to a 0.32 minute increase in EDLOS.

This confirms the results from previous studies [11, 12]. However, the study by Kaushik et al. only investigated the relationship between the TAT and the EDLOS for patients who were being discharged home. We found that the relationship also holds in patients who are admitted to hospital. These patients are probably most likely to benefit from shorter turnaround times, given that they generally have higher acuity levels and their EDLOS was typically longest (data not shown). Furthermore, the TATs in this study were shorter than in other studies, implying that improving the TAT is still worthwhile even if these times are already relatively short.

The low R^2^ (10%) of the fitted model suggests that there are other important factors determining the EDLOS. This is not surprising. An important example of such determinants is the patient’s acuity level [3]. Our study shows that the Emergency Severity Index level, the rate of out-of-range laboratory tests and the destination of the patient after the ED visit, which can be seen as proxies of the acuity level, are indeed positively associated with the EDLOS. Besides acuity level, other studies have found that diagnostic imaging is associated with a longer EDLOS [2, 25]. Even though acuity and the decision to perform diagnostic imaging may have a greater effect on the EDLOS, these factors are not modifiable.

Laboratory turnaround times on the other hand can be modified. For instance, we found that TAT were on average 20.7 and 25.7 minutes shorter in respectively evenings and nights as compared to during the day, most likely due to a lower workload in the clinical laboratory outside of office hours. If this reduction could be realized during office hours, it would lead to an increase in ED capacity of 2.3%. This Is in line with the study by Kaushik et al., which found that a 15 minute reduction in TAT would lead to a 3% increase in ED capacity.

This implies that significant reductions in EDLOS can be achieved through improved TATs. In this study the TAT was further divided into a clinical and a laboratory stage. The laboratory stage is the period from the arrival of the sample at the laboratory until the reporting of the results, thus comprising all the internal processes of the laboratory. It is therefore the stage that is most readily modifiable from the laboratory’s point of view.

Over the last decade, many medical laboratories, including ours, have made substantial progress in improving the TAT. Examples include installing robot track systems for transport of samples to centrifuges and analyzers, using shorter centrifugation times and analyzers with short analytical procedure times, and establishing auto-verification procedures for checking out-of-range results. Such improvements have been shown to reduce the TAT [26–30].

The clinical stage on the other hand has been studied less extensively. This may be due to its multifactorial nature, depending on factors such as the time it takes the ED staff to draw the blood and the logistics of the transport of the sample from the ED to the laboratory, which are influenced by factors as having the tubes labeled with barcodes at the point of care and installing pneumatic tube systems for transport of patient samples.

The TTT is also a potential target for interventions, for instance by pre-ordering laboratory tests and performing the venipuncture immediately after the patient arrives in the emergency department. This is currently standard practice in our hospital, which may be why the median TTT was only 7 minutes in this study.

This study has several limitations. Firstly, this is a single center study in an academic hospital, with a different case mix from general hospitals. Still, this is unlikely to affect the relationship between the TAT and the EDLOS. Secondly, as in all observational studies, there is a risk of unmeasured confounding. For instance, there was no detailed information on the patients’ acuity level, other than the Emergency Severity Index level and the other rather crude proxies mentioned above.

A strength of this study is that it includes all patients coming to the ED who had the aforementioned laboratory tests done. Focusing on this group reduces the risk of confounding that would have been introduced by including all patients with any laboratory testing done, as it is likely that more complicated patients both will have had more laboratory testing done and will have had a longer EDLOS. The selected panel of laboratory tests comprised 62% of all visits during which any laboratory testing was ordered. Another strength is that we were able to divide the TAT in a clinical and a laboratory stage, which can help to determine where potential targets for improvement lie.

## Conclusion

In conclusion, longer time to testing and laboratory turnaround time are associated with a longer emergency department length of stay. However, a causal effect is difficult to determine in this observational setting. Interventions that improve laboratory turnaround times may lead to shorter emergency department lengths of stay, for instance through increased laboratory automation. Prospective studies are needed to investigate whether such interventions affect the adverse patient outcomes associated with ED crowding.

## Data Availability

The datasets used and/or analysed during the current study are available from the corresponding author on reasonable request.

## Abbreviations

CI: confidence interval
ED: emergency department
EDLOS: emergency department length of stay
IQR: interquartile range
SD: standard deviation
TATtu: rnaround time
TTTt: ime to testing
UMC Utrecht: University Medical Center Utrecht
UPOD: Utrecht Patient Oriented Database
